# Evolving blood flow restriction training

**DOI:** 10.1113/EP092255

**Published:** 2025-02-09

**Authors:** Chansol Hurr

**Affiliations:** ^1^ Integrative Exercise Physiology Laboratory, Department of Physical Education Jeonbuk National University Jeonju South Korea

**Keywords:** athletic performance, BFR, exercise training, muscle hypoxia

## 1 INTRODUCTION

Connections link a sequence of three related research papers. The central article which links the other two papers has been published in *Experimental Physiology*. In a Connections article, an author (or authors) of the central article outlines its principal novel findings, tracing how they were influenced by the first article and how the central article has contributed to the developments made in the third article. The author(s) may also speculate on the direction of future research in the field. Connections articles aim to set the research in a wide context.

Blood flow restriction (BFR) training has gained significant recognition for its ability to enhance muscle strength, hypertrophy and endurance, even at lower training intensities. It involves the use of a pneumatic cuff or band around the proximal portion of a limb, which restricts venous outflow while partly occluding arterial inflow, typically using an external pressure of 110–240 mmHg. Recent advancements in BFR research emphasize the need for individualized cuff pressure settings based on arterial occlusion pressure, because fixed pressures can result in inconsistent physiological stimuli across individuals. BFR‐induced occlusion creates a localized hypoxic environment in the exercising muscles, promoting the accumulation of metabolic byproducts, increased recruitment of fast‐twitch muscle fibres and activation of anabolic pathways comparable to those observed with high‐intensity exercise training. BFR training has been shown to induce muscle hypertrophy comparable to that achieved with traditional high‐load resistance training (>70% of one‐repetition maximum), leveraging metabolic stress and growth factor activation as key mechanisms. However, when it comes to maximal strength gains, BFR typically yields smaller improvements, probably owing to reduced mechanical tension, which is a crucial factor for neuromuscular adaptation in high‐load protocols. In contexts where high mechanical loads are contraindicated, such as during recovery from injury or in populations with reduced load tolerance, BFR training provides a practical alternative, offering significant hypertrophic and strength adaptations while minimizing the risk of injury.

Additionally, BFR has shown potential for boosting strength, endurance and sports‐specific performance in athletes. Although some laboratory‐based studies have reported enhancement in performance metrics along with an activation of biochemical pathways associated with angiogenesis and mitochondrial biogenesis (Taylor et al., 2016), real‐world applications of BFR still require further validation to establish performance benefits in well‐trained athletes. Recently, researchers have attempted to modify BFR training to enhance its effects. In the work by Wang et al. (2023), we aimed to incorporate two different approaches into conventional BFR training: (1) maintaining exercise workload during BFR training; and (2) enhancing hypoxic stimulation in the exercising muscles.

Although BFR training can be effective in enhancing athletic performance, power output (or training workload) during BFR training tends to decrease, potentially because of reduced neuromuscular function and delayed muscle phosphocreatine resynthesis. For example, in repeated sprint exercise (RSE) training, the number of sprints and sprint distances decreased with the application of BFR. Considering that the RSE performance is associated with anaerobic glycolytic metabolism, phosphocreatine resynthesis and aerobic metabolism, it is plausible that the continuous application of BFR during RSE training (sprint plus rest) would delay these pathways, leading to reduced power output. The reduced workload observed with continuous BFR appears physiologically appropriate and might not hinder training adaptation, because BFR application is shown to augment skeletal and cardiovascular adaptations. Nevertheless, researchers have focused on refining the BFR modality to maintain the training workload, which could potentially ensure the best training benefits. Kojima et al. (2021) demonstrated that BFR applied only during the rest period of RSE (5–10 s maximal cycling with 40 s rest) leads to greater muscle hypoxia during subsequent sprints, while power output is not compromised when compared with the non‐BFR control conditions (Kojima et al., 2021). Although the muscle hypoxia induced by BFR administered during rest periods was mild in the later phase of RSE (specifically, the last three of five sprints), their findings highlighted a promising modification of BFR application, allowing the training workload to be maintained alongside a BFR‐induced hypoxic stimulus.

Continuous application of BFR in RSE training is presumed to induce greater hypoxia in muscle tissue than BFR applied only during rest periods, owing to the extended duration of blood occlusion. However, limb discomfort associated with continuous BFR is often reported, and some researchers contend that cuff pressure in BFR might not restrict blood flow effectively, as intended, especially during high‐intensity exercise (e.g., RSE), because of dynamic muscle pump action. A new finding by Taylor et al. (2016) was that application of BFR during rest periods of sprint interval exercise (30 s maximal cycling with a 4.5 min rest period) can acutely activate cell signalling pathways related to angiogenesis and mitochondrial biogenesis in muscle tissue, as evidenced by muscle biopsies of the vastus lateralis, potentially enhancing maximal O_2_ uptake in well‐trained cyclists (Taylor et al., 2016). Despite the milder hypoxic stimulus compared with continuous BFR, application of BFR during the rest periods of RSE training appears to be a promising approach for enhancing athletic performance. This is because physiological adaptation does not always scale proportionally to the magnitude of the stimulus. Directly comparing continuous and intermittent BFR in terms of physiological and biochemical outcomes, in addition to long‐term training effects in well‐trained athletes, would provide valuable insights.

**Connected Articles**

- Kojima, C., Yamaguchi, K., Ito, H., Kasai, N., Girard, O., & Goto, K. (2021). Acute effect of repeated sprint exercise with blood flow restriction during rest periods on muscle oxygenation. *Frontiers in Physiology*, *12*, 665383.
- Wang, A., Brothers, R. M., Hurr, C. (2023). Application of blood flow restriction in hypoxic environment augments muscle deoxygenation without compromising repeated sprint exercise performance. *Experimental Physiology*, *108*(5), 728–739.
- Mckee, J. R., Girard, O., Peiffer, J. J., Dempsey, A. R., Smedley, K., Scott, B. R. (2024). Continuous blood flow restriction during repeated‐sprint exercise increases peripheral but not systemic physiological and perceptual demands. *European journal of sport science*, *24*(6), 703–712.

The other approach in our study focused on further enhancing hypoxia in exercising muscles in addition to local hypoxia induced by BFR. A hypoxic environment provides an additional hypoxic stimulus to working muscles via systemic hypoxia, even though the intrinsic mechanisms inducing muscle hypoxia differ (i.e., occlusion‐induced hypoxia vs. limited oxygen availability). Willis et al. (2019) demonstrated that a robust hypoxic stimulus in the muscle tissue could be induced by combining BFR and systemic hypoxia (Willis et al., 2019), which offers promising applicability of BFR in the athletic training field. Our study was influenced by the works of Kojima et al. (2021) and Willis et al. (2019) in terms of maintaining the training workload and delivering a potent hypoxic stimulus. We aimed to fill this gap and demonstrated that application of BFR (140 mmHg) with a combination of systemic hypoxia (inspired oxygen 13.7%) reduced oxygen saturation within exercising muscles in comparison to a single intervention, while maintaining the training workload when BFR was applied during rest periods of RSE (Wang et al., 2023). Specifically, the mild hypoxic stimulus from the BFR during rest, which is often considered a limitation, could be augmented by applying systemic hypoxia in a low‐oxygen environment, thereby achieving more intense hypoxia in exercising muscles without reducing power output. We also observed that the maintained power output in the combined condition (BFR plus systemic hypoxia) during RSE might be attributable, in part, to increased neuromuscular activation, as indicated by surface EMG data. It should be noted that a constant cuff pressure (140 mmHg) was applied in our study owing to logistical constraints, specifically the limited 30 s application window within a 60 s rest period. Considering the inter‐individual variability observed, the adoption of individualized cuff pressure settings (e.g., a percentage of arterial occlusion pressure) is strongly recommended for future BFR research, where feasible.

Recently, McKee et al. (2024) investigated the effects of continuous BFR (rest plus sprint) during a multiset of RSE training on performance and other physiological parameters (McKee et al., 2024). Their study incorporated more practical implications than previous BFR research, including our own. For example, considering that sprint durations in team sports typically range from 2 to 3 s, their RSE protocol included three sets of five 5 s cycling sprints, with a 25 s rest between repetitions and a 3 min rest between sets. This RSE protocol also reflects a practical training regimen for team‐sport athletes, who typically engage in closed‐loop multiset training. The authors demonstrated that BFR administered during RSE training led to greater muscle deoxygenation (i.e., stronger hypoxic stimulus) and decreased power output, consistent with the findings from previous studies applying continuous BFR. The work by McKee et al. (2024) is considered to have refined the RSE training protocol and underscored the practical applicability of BFR in athletic training.

Although BFR training demonstrates promise as an effective training modality, further research is essential to delineate its comparative effectiveness against traditional training, particularly in enhancing specific performance metrics, such as time trials in well‐trained athletes. With ongoing refinements, BFR could become a key component of athletic conditioning, enabling precise and effective muscle adaptation across diverse sporting disciplines. I hope that this compelling area continues to advance, leading to increasingly refined and widely applied BFR practices across various athletic disciplines.

## AUTHOR CONTRIBUTIONS

Sole author.

## CONFLICT OF INTEREST

None declared.
